# Indoor concentrations of particulate matter 2.5 in a pediatric emergency service

**DOI:** 10.1590/1984-0462/2022/40/2020330

**Published:** 2021-10-04

**Authors:** Catiane Zanin Cabral, Alan da Silveira Fleck, Fernanda Chaves Amantéa, Claudia Ramos Rhoden, Sérgio Luis Amantéa

**Affiliations:** a Universidade Federal de Ciências da Saúde de Porto Alegre, Porto Alegre, RS, Brazil.

**Keywords:** Air pollution, Emergency medical services, Particulate matter, Poluição do ar, Serviços médicos de emergência, Material particulado

## Abstract

**Objective:**

To evaluate air quality in the waiting room of a pediatric emergency service considering the serial concentrations of particulate matter (PM_2.5_), and to determine if the number of people present in the room can have an influence on the pollutant concentrations.

**Methods:**

Cross-sectional study, carried out in the waiting room of a reference pediatric hospital in the city of Porto Alegre, conducted in a one-year period, in a continuous-time sample including all of the four seasons of the year. The monitoring of PM_2.5_ was performed using a real-time aerosol monitor (DustTrak II). The number of people in the room was determined every hour and the climatic characteristics per daily mean. The concentration of PM2.5 and the number of people were expressed by mean and standard deviation. The means were compared by Analysis of Variance and Pearson's correlation coefficient.

**Results:**

There was a significant increase in the concentration of PM_2.5_ in the autumn, when compared to other seasons (p<0.001). The pollutant increase, in this season, was accompanied by the higher number of people in the emergency room (p=0.026). The association between PM_2.5_ and the number of people is confirmed by the positive correlation between these two variables (r=0.738; p<0.001).

**Conclusions:**

The pediatric emergency waiting room showed elevated PM_2.5_ in all seasons. The number of people in the room had a positive correlation with the concentration of the pollutant in the environment.

## Resumo

**Objetivo:**

Avaliar a qualidade do ar na sala de espera de um serviço de emergência pediátrica pelas concentrações ambientais seriadas de material particulado (MP_2,5_) e determinar se o número de pessoas presentes no ambiente influencia as concentrações do poluente.

**Métodos:**

Estudo transversal, realizado na sala de espera de um hospital pediátrico de referência na cidade de Porto Alegre, conduzido ao longo de um ano, com um período de amostragem temporal contínua com duração de uma semana, em todas as quatro estações do ano. O monitoramento do MP_2,5_ foi realizado por meio de um monitor de aerossol em tempo real (DustTrak II). O número de pessoas no ambiente foi determinado a cada hora e as características climáticas por média diária. As concentrações de MP_2,5_ e o número de pessoas foram expressos por médias e desvio padrão. As médias foram comparadas por análise de variância e pelo coeficiente de correlação de Pearson.

**Resultados:**

Houve aumento significativo na concentração do MP_2,5_ no outono, quando comparado com as outras estações (p<0,001). O aumento desse poluente, nessa estação, esteve acompanhado de maior número de pessoas na emergência (p=0,026). A associação entre MP_2,5_ e número de pessoas é ratificado pela correlação positiva entre essas duas variáveis (r=0,738; p<0,001).

**Conclusões:**

A sala de espera da emergência pediátrica apresentou concentrações elevadas de MP_2,5_ durante todas as estações do ano. O número de pessoas no ambiente guardou correlação positiva com as concentrações do poluente no ambiente.

## INTRODUCTION

It is observed that the exposure of the population to air pollutants can be higher in closed spaces, in comparison to the outdoors. Some studies have shown that the particulate matter 2.5 (PM_2.5_) has presented higher mean concentrations indoors than outdoors. The individual contact with such a pollutant indoors can be a result of the exposure to several sources. This type of pollution is usually composed of products brought in by people in their shoes, clothes and utensils; besides, it can be carried through the wind when doors and windows are open.^1^ ^-^ ^3^ It has been attracting more interest in the research field, considering that people who are exposed to a polluted indoor environment can also be more prone to acquiring diseases.^4^

In this context, the evaluation of air quality in hospitals has been systematically presented due to its peculiarities. In a pediatric hospital, it can be even more interesting, considering the higher number of professionals involved in patient care and the need for an accompany person (parents and/or relatives) in the hospital environment.^5^ ^,^ ^6^ In this situation of more traffic coming from the external areas, there could be an increase in the presence of microorganisms and particles in the air.^7^ ^,^ ^8^ From the point of view of care, such an exposure in overcrowded hospital rooms has the potential to impact morbidity and, consequently, even influence hospital costs.^3^

Recently, with the advent of the Coronavirus pandemic, this topic gained more relevance. Guo et al. tried to identify the presence of SARS-CoV-2 in Hospital Huoshenshan, in Wuhan (China) during the epidemic. They were able to show the presence of the agent in the air and found high rates of its presence even on the floor (up to 70%) and shoe soles (up to 50%). They established the hypothesis that the particles suspended in the air would be deposited on the ground due to gravity, and could be carried by the feet to areas without patients.^9^

This scenario makes it so that the particulate matter is one of the most critical markers of air quality, both indoors and outdoors. Its respirable fraction (2.5 μm) is able to reach the small airway caliber and promote alveolar deposition; besides, it is associated to the higher prevalence of several respiratory, cardiovascular and metabolic diseases conditions.^10^ Therefore, we proposed to evaluate if air quality in the waiting room of an emergency pediatric service is influenced by the number of people circulating in the room.

## METHOD

This is a cross-sectional study aiming at identifying the concentration of PM_2.5_ in the air of the waiting room in the emergency department of Hospital da Criança Santo Antônio (pediatric hospital). The institution is located in the central area of the capital (Porto Alegre) of the State of Rio Grande do Sul (30°1’49.65”S/51°13’11.59”O). The physical structure of the emergency department is in the corner of two pathways with intense automotive flow. One of them is capable of registering a circulation of up to 3,539 cars/hour (Public Company of Transport and Circulation — EPTC). The waiting room of the pediatric emergency has an area of 60 m^2^, is 2.80 m high, has four large glazed areas and a single door to control flow (entrance/exit). The physical area is climatized by a central air equipment with a partial recirculation system, without any special filters.

The environmental samples of PM_2.5_ were collected in the first weeks of the four seasons of the year, throughout one year: March (22 to 28 – autumn), June (23 to 29 – winter), September (24 to 30 – spring), January (3 to 9 – summer).

PM_2.5_ was monitored inside the waiting room of the pediatric emergency service, and the equipment was placed 150 cm above the ground in a protected area. We used a real-time aerosol monitor (DustTrak II^®^ Model 8532, ETI Incorporated, St. Paul, MN, USA) equipped with an impactor that allows the entry of particles with less than 2.5 μm in aerodynamic diameter. These particles are transported to an optical chamber with an infrared light beam that, through light dispersion, provides the real-time measurement of particles. The airflow to be measured was programmed for 3 L/min. The equipment was programmed to register the mean concentration of PM_2.5_ per minute.

The sampling period was 24 hours during a single week of each one of the four seasons of the year. The meteorological conditions in the air sampling periods were observed at the Institute of Meteorology of Porto Alegre.

The mean of the total number of people in the waiting room was obtained through the hourly counting of the number of people in the room from the point of view of the administration desk (located at a lateral position, with total view of the waiting room). In a standardized form, an employee registered the number of patients and the accompany persons in the waiting room in fixed 60-minute intervals. At the end of the day, the sum of the number (patients + accompany persons) in the records was divided by the number of hours (24 hours).

The concentration of PM_2.5_ and the number of people in the room were expressed in a descriptive manner through means and standard deviation.

The comparison between means was carried out by the analysis of variance (one-way ANOVA), followed by the Bonferroni post hoc test. The Kolmogorov-Smirnov test was applied to verify the normality of the distribution of variables. The association between continuous variables was performed using the Pearson's correlation coefficient. Significance level was 5% (p≤0.05), and the analyses were made using the Sigma Plot software, version 12.0.

## RESULTS

The daily mean concentration of PM_2.5_ in each season of the year, as well as the number of people present at the hospital emergency room during the monitoring period and the meteorological conditions are presented in Table 1.

**Table 1:** Descriptive analysis of the concentration of particulate matter 2.5, number of people and meteorological conditions in each season of the year.

Season	Days	PM (μg/m^3^)	N. of people per hour	Mean temperature (°C)	Humidity (%)	Rain (mm)
Spring	Day 1	63.1	33.4	24.1	71.5	0.0
	Day 2	34.1	31.5	24.4	65.8	1.5
	Day 3	10.3	27.7	22.7	67.8	0.0
	Day 4	10.2	23.8	26.3	61.5	0.0
	Day 5	12..6	19.8	24.9	79.3	4.0
	Day 6	27.4	17.5	21.1	73.5	9.3
	Day 7	25.9	18.5	20.9	62.0	0.0
Summer	Day 1	14.8	28.1	22.1	62.8	0.0
	Day 2	13.4	10.4	22.4	61.3	0.0
	Day 3	18.4	14.2	23.7	69.3	0.0
	Day 4	15.3	13.2	24.9	66.8	0.0
	Day 5	8.7	15.3	24.8	68.3	0.0
	Day 6	8.8	12.1	25.6	71.5	0.0
	Day 7	18.4	11.5	26.2	68.3	0.0
Autumn	Day 1	47.2	40.1	16.0	86.3	0.0
	Day 2	65.4	33.3	15.5	84.4	0.0
	Day 3	40.9	35.9	14.8	83.4	0.2
	Day 4	54.5	36.4	14.7	76.4	0.0
	Day 5	51.7	28.4	16.1	78.8	0.0
	Day 6	64.4	28.5	15.3	89.3	3.0
	Day 7	74.3	34.9	15.7	85.3	0.0
Winter	Day 1	48.0	31.2	19.4	79.0	6.5
	Day 2	17.6	18.2	12.0	92.0	2.6
	Day 3	13.4	24.8	10.8	96.0	22.6
	Day 4	23.1	12.5	11.2	82.0	16.9
	Day 5	47.9	27.7	11.8	79.0	0.1
	Day 6	26.3	19.9	12.4	88.0	0.8
	Day 7	18.0	25.5	9.3	87.0	3.6

PM: particulate matter.

**Figure 1:**
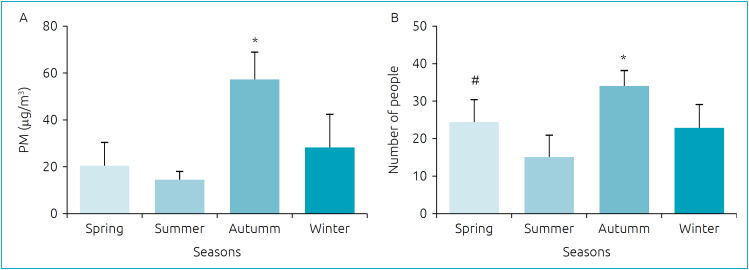
Concentration of particulate matter 2.5 and number of people in the waiting room of the emergency service of a pediatric hospital in the four seasons of the year. (A) Mean of particulate matter 2.5 in each season: *autumn is significantly higher than all of the other seasons (p<0.001). (B) Mean number of people in the emergency room in each season: *autumn is significantly higher than all other seasons (p=0.026). ^#^spring is significantly higher than summer (p=0.041).

As demonstrated in Figure 1A, there was a significant increase in the concentration of PM_2.5_ in the autumn, when compared to other seasons (p<0.001). The increase in this pollutant, in this season, was accompanied by the increment in the number of people in the emergency room (p=0.026) (Figure 1B).

**Figure 2:**
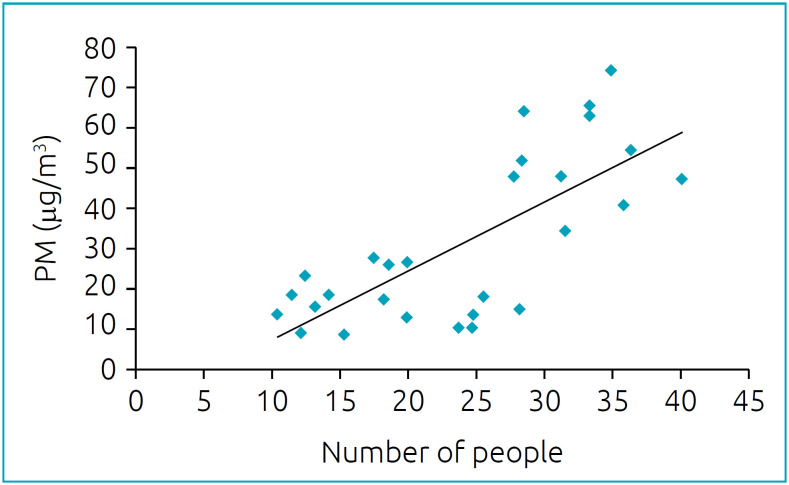
Correlation between the concentration of particulate matter 2.5 and the number of people in the waiting room of the emergency service of a pediatric hospital in the four seasons of the year. There was a significant positive association between the mean number of people per day and particulate matter (r_p_=0.738; p<0.001). This association remained significant regardless of temperature, humidity and rainfall (_partial_r=0.651; p=0.001).

The symmetrical behavior between PM_2.5_ and the number of people registered is proven by the positive correlation between these two variables, as shown in Figure 2 (r=0.738; p<0.001). This correlation is maintained even when adjusted for the possible influence of meteorological variables, such as mean temperature, humidity, and rainfall (r=0.651; p=0.001).

In an isolated manner, the number of people present in the waiting room ranged according to the season of the year (p<0.05). In the summer, season that presented the lowest number of people in the room, the mean number was 15.0±5.5 people per hour, whereas in autumn, season that presented the highest number of people, the mean was 33.9±3.9 people per hour. Winter and spring presented, respectively, 22.8±5.8 and 24.15±5.8 people per hour.

## DISCUSSION

The results of this study demonstrate that autumn was the season with the highest concentrations of PM_2.5_ in the waiting room of the pediatric emergency service. Such concentrations of PM_2.5_ were higher than those established by the World Health Organization, referring to the daily mean of 25 μg/m³ for this pollutant.^11^ We also found an association between the concentrations of PM_2.5_ in the air and the increment in the number of people present in the studied room.

Several factors, including the location in the hospital, temperature, relative humidity in the air, rainfall and the number of people circulating were controlled. In this investigation, the number of occupants in the waiting room was positively correlated to the concentrations of PM_2.5_, referring to the measurements taken in the four seasons of the year. Our result corroborates the data by Tang et al.^12^, when they studied an intensive care unit (ICU) in Taiwan and verified a positive association between the concentration of particles and the number of people circulating in the hospital environment. In that study, the concentration of CO_2_, PM_10_ and coarse particles was higher after the visitation period to the patient, when compared to calmer periods, which leads to the conclusion that visits to patients had a negative impact on the internal atmosphere of the ICU.

Indoor air quality can be influenced by the internal sources of pollution, including the habits of individuals who circulate and the poor planning of the facilities.^13^ Thus, the levels of PM_2.5_ matter in the room can range according to the type and number of activities performed in each location. Specifically regarding hospital environments, previous studies have shown that the indoor air quality depends on the number of present people, the flow of individuals and the quality of the ventilation system.^12^ ^-^ ^14^

Another factor that may have an indirect contribution in the high concentration of PM_2.5_ in the room is the resuspension phenomenon. Experimental studies have shown the capacity of the particulate matter deposited by gravity to be resuspended and return to the air.^15^ ^-^ ^18^ In this case, the number of people circulating in the room and the contact of the shoe sole with the floor can be factors that justify the higher concentrations of particulate matter in the room.

The recent COVID-19 epidemic has been a reason for major concerns considering this possibility, because of the potential for contamination. Guo et al. were the first to consider it, once they identified the presence of SARS-CoV-2 both in the air and in solid surfaces, including floors and shoe soles.^9^

Some limitations should be mentioned in our study. The most significant one is that it was not possible to measure the outdoor concentrations of PM_2.5_ during the monitoring periods, nor the characteristics of wind dispersion (velocity and direction). Therefore, it is not possible to determine if the outdoor concentration of PM_2.5_ may have had a direct influence on the indoor levels we found. However, this fact does not stop us from establishing the conclusion that the concentrations of PM_2.5_ in the room are influenced by the number of people in the waiting room of the pediatric emergency service.

The overcrowded emergency services are a global problem and can be expressed by the increasing use of the services in rates that are 65% higher than those of the growth of the American population. Such a phenomenon results in the reduction of care quality, care-related risks and, as we could observe, even environmental risks. The attempts to improve the process go through the discussion of improved flows, use of protocols, structure of screening tools, informatization of processes and adequate sizing of work force, among others.^19^ ^,^ ^20^ Aspects related to the structure of waiting rooms do not follow a universal pattern, nor are they emphasized by the literature. In our country, a policy to implement emergency care units (UPA) has established some rules related to the physical structure of the waiting room. These sectors should have an area between 24 and 72m^2^ and try to meet the minimum sizing of 1.2 m^2^/person.^21^

Just as the literature has shown an association between the overcrowding and damage to care, we can observe that the saturation of the physical waiting room in an emergency service can cause a negative impact on indoor air quality.
